# Chloroplast genome structure and phylogenetic position of *Lophatherum gracile*

**DOI:** 10.1080/23802359.2020.1845577

**Published:** 2021-01-06

**Authors:** Caibi Zhou, Xiaolu Zhou, Tingrui Dai, Zhirui Wen, Xiying Guo, Yuyan Song, Lu Long, Yaling Li, Xin Mei

**Affiliations:** a College of Biological Science and Agriculture, Qiannan Normal University for Nationalities, Duyun, China; b College of Life Sciences, Gannan Normal University, Ganzhou, China

**Keywords:** *Lophatherum gracile*, Illumina sequencing, chloroplast genome, phylogenetic position

## Abstract

*Lophatherum gracile* is distributed in south China, Japan and South Asia, and it is wild in the valley, stream, woodland, forest edge and gully edge. In this study, the complete chloroplast genome sequence of *Lophatherum gracile* was successfully obtained using Illumina sequencing. The full length of the chloroplast genome length was 137,749 bp with a typical quadripartite structure: one large single copy (LSC) region (80,610 bp), one small single copy (SSC) region (12,429 bp), and a pair of inverted repeats (IRs) (22,355 bp each). The GC content of this genome was 38.64%. The whole genome contained 130 genes, including 85 protein-coding genes, 37 tRNA genes, and 8 rRNA genes. Phylogenetic analysis indicated that *Lophatherum gracile* was closely related to *Cenchrus americanus* and *Cenchrus longispinus*.

*Lophatherum gracile* is distributed in the south region of the Yangtze River in China, as well as in India, Sri Lanka, Myanmar, Malaysia, Indonesia, new guinea island and Japan, and it is wild in the valley, stream, woodland, forest edge and gully edge (Institute of Drug Control & South China Institute of Botany 1963; Editorial Committee of flora of China 2002). *Lophatherum gracile* belongs to *Gramineae* family and is a perennial herb. Its dried stems and leaves can be used for medicine to clinically clear heat, disinhibit dampness, and treat inflammation (Editorial Committee of flora of China 2002; Y. Chen 2014; Wang et al. 2016; Lai et al. 2021). Its leaves are rich in flavonoids, phenolic acids and polysaccharides (Xue et al. 2009; Zhang 2010; Tang et al. 2012;). Through modern pharmacological analysis, it is found that *Lophatherum gracile* has antibacterial, anticancer, anti-aging and diuretic effects (M. Chen et al. 1999; Liu 2008; Xue et al. 2009; Huang and Cronk 2015; Istiqomah et al. 2015). It can protect liver and myocardium (Kim et al. 2009; Lin et al. 2010; Fu and Chen 2013). The ethanol extract of *Lophatherum gracile* has antiviral activity against respiratory syncytial virus infection (L. Chen et al. 2019). To identify *Lophatherum gracile* accurately and then guarantee the medicinal quality, herein the complete chloroplast genome of *Lophatherum gracile* was sequenced by using next-generation technology to study the taxonomy for *Lophatherum gracile*.

The leaves of *Lophatherum gracile* were collected from Tea Garden of Qiannan Normal University for Nationalities (26.28°N, 107.47°E) in Duyun City, Guizhou Province, China. The specimen (DZ20200608) was deposited in the herbarium of Qiannan Normal University for Nationalities. Total DNA was extracted from fresh leaves using a CTAB method and then sequenced using the Illumina Novaseq platform. Low-quality reads and adapters were trimmed off using the FastQC software (Andrews 2010), and genome was de novo assembled using SPAdes v3.9 (Bankevich et al. 2012), and then annotated using Plann software (Huang and Cronk 2015). The complete chloroplast genome was submitted to the GenBank database (Accession Number: MT872396).

The chloroplast genome size of *Lophatherum gracile* was 137,749 bp with four typical sub-regions: a large single copy (LSC) region of 80,610 bp, a small single copy (SSC) region of 12,429 bp, and two inverted repeats (IRs) containing 22,355 bp each. The guanine-cytosine (GC) content in this genome was 38.64%. The whole genome contained 130 genes, including 85 protein-coding genes, 37 tRNA genes, and 8 rRNA genes. Twenty one genes were duplicated in the IRs regions, which were 9 protein-coding genes (rps19, rpl2, rpl23, ndhB, rps7, rps12, ycf68, ycf1, rps15), 8 tRNA genes (trnH-GUG, trnl-CAU, trnL-CAA, trnV-GAC, trnl-GAU, trnA-UGC, trnR-ACG, trnN-GUU), and 4 rRNA genes (rrn16S, rrn23S, rrn4.5S, rrn5S).

To analyze the phylogenetic position of *Lophatherum gracile*, 17 complete chloroplast genomes from the same *Gramineae* family were downloaded from the GenBank database, and these genomes were aligned using MAFFT (Katoh and Standley 2013) and the phylogenetic tree was constructed using Mega-X v10.0.5 software (Kumar et al. 2018) with maximum likelihood method and 1000 bootstrap replicates (Figure 1). The phylogenetic tree shows that *Lophatherum gracile* formed a distinct clade which was separated from *Cenchrus americanus* and *Cenchrus longispinus*. These results facilitate the identification of *Lophatherum gracile* and its taxonomic study, which will help us collect resources, culture them, study their pharmacological activities, and develop some functional products.

**Figure 1.**
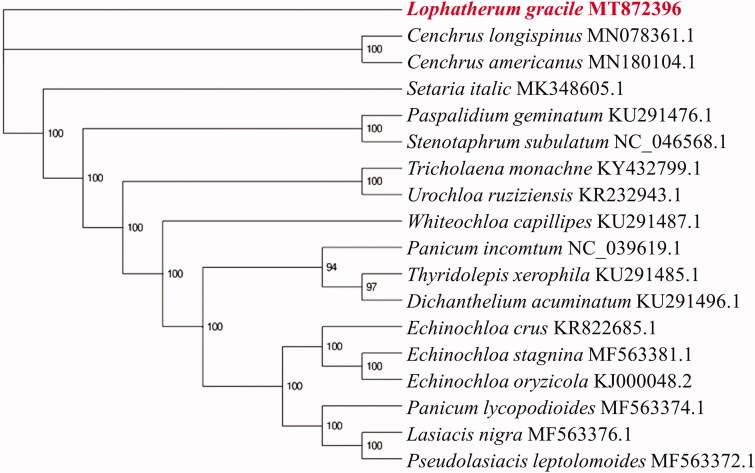
The maximum likelihood (ML) phylogenetic tree of 18 *Gramineae* chloroplast genomes.

## Data Availability

The data that support the findings of this study are openly available in GenBank of NCBI at https://www.ncbi.nlm.nih.gov, reference number MT872396.
